# Understanding Electrodeposition of Chitosan–Hydroxyapatite Structures for Regeneration of Tubular-Shaped Tissues and Organs

**DOI:** 10.3390/ma14051288

**Published:** 2021-03-08

**Authors:** Katarzyna Nawrotek, Jacek Grams

**Affiliations:** 1 Department of Environmental Engineering, Faculty of Process and Environmental Engineering, Lodz University of Technology, Wolczanska 213 Street, 90-924 Lodz, Poland; 2 Institute of General and Ecological Chemistry, Faculty of Chemistry, Lodz University of Technology, Zeromskiego 116, 90-924 Lodz, Poland; jacek.grams@p.lodz.pl

**Keywords:** electrodeposition, biomaterials, tissue regeneration, implants, chitosan, hydrogel

## Abstract

Tubular-shaped hydrogel structures were obtained in the process of cathodic electrodeposition from a chitosan–hydroxyapatite solution carried out in a cylindrical geometry. The impact of the initial concentration of solution components (i.e., chitosan, hydroxyapatite, and lactic acid) and process parameters (i.e., time and voltage) on the mass and structural properties of deposit was examined. Commercially available chitosan differs in average molecular weight and deacetylation degree; therefore, these parameters were also studied. The application of Fourier-transform infrared spectroscopy, scanning electron microscopy, and time-of-flight secondary ion mass spectrometry allowed obtaining fundamental information about the type of bonds and interactions created in electrodeposited structures. Biocompatible tubular implants are highly desired in the field of regeneration or replacement of tubular-shaped tissues and organs; therefore, the possibility of obtaining deposits with the desired structural properties is highly anticipated.

## 1. Introduction

The electrophoretic deposition (EPD) method is attracting attention due to its versatility, low cost, and capability to form a variety of composite films. It has been applied in many fields, e.g., biology, biotechnology, biochemistry, chemistry, and materials science [1,2,3,4,5].

Two phenomena can be distinguished in EPD: electrophoresis and deposition. The former one, electrophoresis, encompasses the motion of charged particles in a liquid medium toward an electrode evoked by the application of an electric field. In the latter one, deposition, cations or anions deposit on an oppositely charged electrode and create films or monoliths. The control over process parameters, as well as solution composition, allows obtaining deposits with desired microstructural homogeneity and thickness [6]. These structural features are critical for tissue engineering, which is focused on materials able to biomimic structurally lost tissues and organs. EPD has been used to obtain multifunctional coatings exhibiting strong bonding ability to desired tissues, fabricate three-dimensional scaffolds, or make scaffolds bioactive [6,7,8]. The most widely used materials are natural polymers and macromolecules. One of the polyelectrolytes successfully applied in EPD is chitosan [9].

Chitosan is a partially deacetylated derivative of chitin. Partial deacetylation of chitin leads to the formation of polymer composed of randomly distributed β-(1→4)-linked d-glucosamine (deacetylated unit) and N-acetyl-d-glucosamine (acetylated unit). Commercially available chitosan differs with average molecular weight and deacetylation degree. These parameters affect the interaction of chitosan macromolecules with other organic and inorganic reagents. Chitosan is soluble only in some organic acids (e.g., formic, acetic, propionic, lactic, citric, and succinic acid) and in very few inorganic acids (e.g., hydrochloric, phosphoric, and nitric acid) [10,11,12]. Its solubility depends on the value of pKa of acid and its concentration [13]. Chitosan is soluble at dilute acidic solutions below pH = 6.0–6.5 [14]. At low pH, the amino groups of chitosan molecules get protonated. This results in a positively charged polymer chain. When pH exceeds 6.0, protonated amino groups release protons and hydrophobicity along the polymer chain increases. This soluble–insoluble transition takes place at pKa value of 6.3. The pKa strongly depends on the deacetylation degree and the method of chitin deacetylation [15]. Furthermore, the solubility of chitosan is strongly affected by polymer molecular weight (MW), whereby higher MW results in less solubility [13]. In addition, the concentration of protons needed to dissolve chitosan must be at least equal to the concentration of –NH_2_ units involved. On the other hand, –NH_2_ units are responsible for creation of bonds and interactions with other elements and groups. For example, chitosan is known for its good complexing ability of metals.

Peng and Zhitomirsky [16] employed electrophoretic deposition in order to prepare composite coatings from chitosan solutions. They performed studies correlating a chitosan film growth rate as a function of pH and polymer concentration. The electrodeposition was performed from suspensions of hydroxyapatite and chitosan in ethanol–water solvent. The work of Geng and colleagues [17] was focused on developing a new electrodeposition method for the production of chitosan hydrogel films with various shapes based on the coordination of chitosan to the metal ions generated in situ by simultaneous electrochemical oxidation. The obtained hydrogel deposit had a smooth and homogeneous surface. Moreover, it possessed sufficient strength to be peeled from the electrode. In 2016, our team reported electrophoretic deposition from chitosan–hydroxyapatite acidic solutions [18]. The novelty of this approach was the use of a rod mandrel as a cathode. The applied cylindrical geometry allowed obtaining tubular-shaped hydrogel structures. Despite the promising potential of electrodeposited chitosan-based implants in the field of regeneration or replacement of cylindrical tissues and organs, there is little known about the nature of interactions between their constituents. It seems that, by studying the influence of initial concentration of solution components, as well as process parameters, on the resulting mass and structural properties of deposits, the knowledge considering the type of chemical interactions created in the process of electrodeposition from chitosan–hydroxyapatite solutions may be gathered.

It is hypothesized that electrodeposition from an acidic solution of chitosan and hydroxyapatite leads to co-deposition of chitosan and calcium-based moieties from hydroxyapatite. In this work, the electrophoretic deposition mechanism from chitosan–hydroxyapatite solutions was studied in a cylindrical geometry. The application of such a geometry allowed obtaining tubular-shaped deposits, which may find application in the replacement of tubular-shaped tissues and organs. The aim of this work was to assess the relevance of some parameters, such as the initial concentration of solution components (i.e., chitosan, lactic acid, and hydroxyapatite) and process conditions (i.e., time and voltage), to the performance of the electrophoretic deposition. The mass of deposits was evaluated as a function of the studied parameters. In addition, structural characteristics of chitosan were considered (i.e., molecular weight and degree of deacetylation). Fourier-transform infrared spectroscopy (FTIR), scanning electron microscopy (SEM), and time-of-flight secondary ion mass spectrometry (ToF-SIMS) were applied in order to study structural characteristics of deposits.

## 2. Materials and Methods

### 2.1. Materials

Chitosan (CH, type: 85/100, 85/500, 85/1000, 75/500, and 95/500) was acquired from Heppe Medical Chitosan GmbH (Halle, Germany). The degree of deacetylation (DD), viscosity (µ, 1% in 1% acetic acid, 20 °C), and viscosity average molecular weight (Mv) for the polymers are listed in Table 1. Mv was determined by viscometry [19]. Lactic acid (LA) and hydroxyapatite (HAp, nanopowder, <200 nm particle size) were purchased from Merck KGaA (Darmstadt, Germany).

### 2.2. Implant Manufacturing

A fabrication method of implants based on electrophoretic deposition from chitosan solution was previously developed in our laboratory [18]. Briefly, lactic acid was dissolved in deionized water. Then, chitosan and hydroxyapatite were added. The obtained solution was stirred (under slow rotations) until complete dissolution for 24 h. Next, 22 mL of the solution was poured into the tank of a specially designed reactor. The inner electrode (i.e., cathode) applied had an outside diameter of 2 mm and the outer one (i.e., anode) had an inside diameter of 30 mm. The electrodeposition process was conducted at room temperature. Table 2 lists the parameters studied in this work (chitosan concentration (CH 85/500), lactic acid concentration, hydroxyapatite concentration, voltage, and time). Moreover, the influence of structural characteristics of chitosan was studied (i.e., molecular weight and degree of deacetylation (Table 1)). The applied protocol allowed producing a 38 mm long conduit. Following the fabrication process, the obtained structures were gently removed from the inner electrode and subjected to chemical, physical, and biological assessment.

### 2.3. Structural Studies

Samples for dry mass, SEM, FTIR, and ToF-SIMS analyses were taken immediately after fabrication and exposed to a drying procedure. Firstly, deposits were dehydrated by placing in an exsiccator containing water and ethyl alcohol (1:1) for 24 h. Then, the samples were removed from the exsiccator and left open for 24 h to evaporate the ethanol. Such a drying protocol allowed keeping the tubular shape of deposits.

#### 2.3.1. Implant Morphology—SEM

Scanning electron microscopy photographs of gold-coated cross-sections and longitudinal views of conduits were taken with a Hitachi TM-1000 microscope (Hitachi, Ltd., Tokyo, Japan). Dry conduits were cut into specimens of length 5 ± 0.2 mm for examination.

#### 2.3.2. Implant Structure Characterization—FTIR and ToF-SIMS

The manufactured conduits were analyzed using a Nicolet™ iS50 FTIR Spectrometer (Thermo Scientific, Waltham, MA, USA) equipped with a diamond ATR (attenuated total reflection). The spectra were collected over the wavelength range of 500–4000 cm^−1^.

ToF-SIMS measurements were performed using a TOF-SIMS IV spectrometer (ION-TOF GmbH, Münster, Germany) equipped with a 25 kV pulsed Bi^3+^ primary ion gun in the static mode. For each sample, at least three spectra were collected from different locations. The analyzed area corresponded to a square of size 100 × 100 µm^2^. A pulsed electron flood gun was used for charge compensation. The samples were fixed to the sample holder using adhesive tape. The mass spectra were calibrated using $H^{+}$, ${\mathrm{CH}}_{3}^{+}$, and $C_{2}H_{5}^{+}$ ions.

#### 2.3.3. Water Content

In order to determine the water content of hydrogels, the deposits were divided into two groups: hydrated and dry. The mass of hydrated specimens was measured just after their preparation. To determine the mass of dry ones, deposits were placed at 70 °C for 12 h. A mean value of three different measurements for both measurements was determined. The water content was calculated using the following equation:

(1) $$Χ=\frac{m_{h}-m_{d}}{m_{h}}\times 100\%,$$

where Χ is the water content (%), m_h_ is the mass of hydrated deposit (g), and m_d_ is the mass of dry deposit (g).

## 3. Results

**Figure 1:**
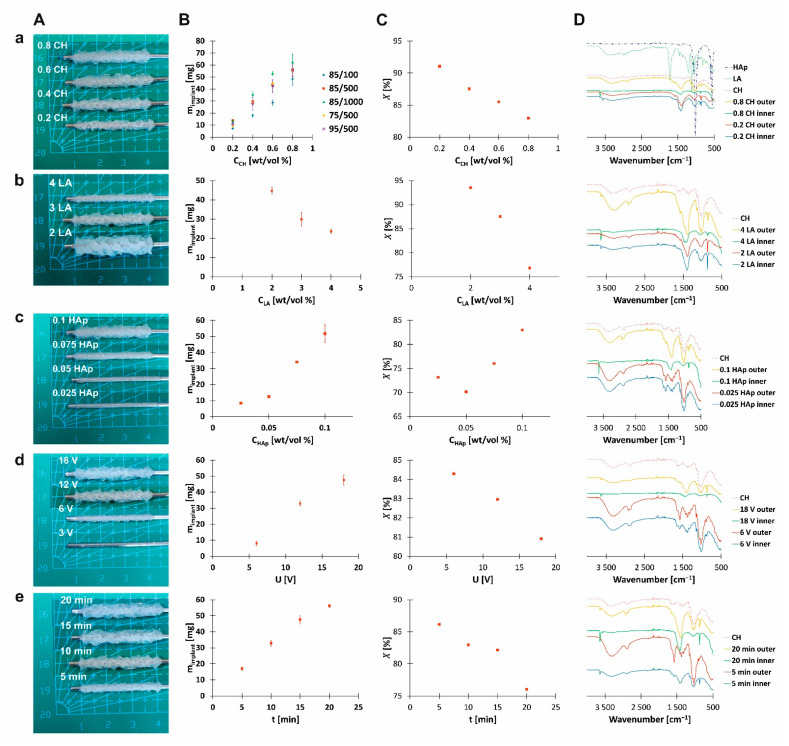
The influence of (**a**) chitosan concentration, (**b**) lactic acid concentration, (**c**) hydroxyapatite concentration, (**d**) voltage, and (**e**) time on (**A**) characteristics, (**B**) dry mass, (**C**) water content, and (**D**) Fourier-transform infrared (FTIR) spectra of tubular-shaped chitosan–hydroxyapatite deposits. The solution concentrations are listed in Table 2.

**Figure 2:**
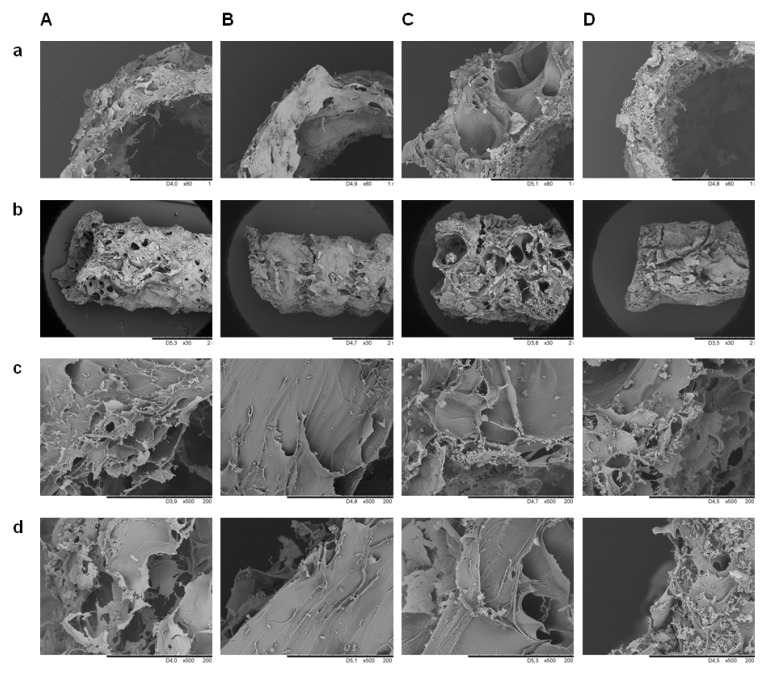
SEM photographs of deposits obtained from solutions containing (**A**) 0.4 wt./vol.% CH, 0.05 wt./vol.% HAp, 3 wt./vol.% LA (10 min), (**B**) 0.6 wt./vol.% CH, 0.05 wt./vol.% HAp, 3 wt./vol.% LA (10 min), (**C**) 0.6 wt./vol.% CH, 0.1 wt./vol.% HAp, 3 wt./vol.% LA (10 min), and (**D**) 0.4 wt./vol.% CH, 0.1 wt./vol.% HAp, 3 wt./vol.% LA (30 min). (**a**) Transverse cross-section, (**b**) longitudinal view, (**c**) 200× magnification of inner surface, and (**d**) 200× magnification of outer surface.

In the performed electrophoretic deposition from chitosan–hydroxyapatite solutions, deposits were obtained at the rod-shaped cathode (Figure 1A and Figure 2). The deposition yield was dependent on the initial concentration of solution components (i.e., chitosan, lactic acid, and hydroxyapatite), as well as process parameters (i.e., time and voltage) (Figure 1B). Moreover, the structural characteristics of chitosan (i.e., molecular weight and degree of deacetylation) also influenced the weight of deposits (Figure 1Ba). Nearly linear dependences were obtained between the mass of deposit and all studied parameters, indicating the constant deposition rates.

The images of implants prepared from chitosan–hydroxyapatite solutions differing in concentration of chitosan (i.e., maintaining a constant concentration of lactic acid and hydroxyapatite) are shown in Figure 1Aa. A higher chitosan concentration led to a higher mass of deposit (Figure 1Ba) and a lower water content (Figure 1Ca). In addition, a higher molecular weight of chitosan chains led to a higher mass of deposit (Figure 1Ba). The deacetylation degree of chitosan had an insignificant influence on the resulting deposit mass (Figure 1Ba). The water content measured for the implant prepared from solutions with a concentration of CH equal to 0.8 wt./vol.% CH for chitosan with the same degree of deacetylation but differing in viscosity average molecular weight, i.e., CH 85/100, CH 85/500, and CH 85/1000, was equal to 77.06%, 82.96%, and 82.25%, respectively, whereas, for chitosan with the same viscosity average molecular weight but differing in degree of deacetylation, i.e., CH 75/500, CH 85/500, and CH 95/500, the water content was equal to 84.55%, 82.96%, and 77.31%, respectively. The SEM images show that a higher chitosan concentration resulted in less porous and more compacted structures (Figure 2A,B), corresponding to the water content studies. The electrodeposition was accompanied by significant gas evolution, which was a reason for the porosity of deposits.

In this work, it was assumed that chitosan was a base component of the obtained structures; therefore, the FTIR analysis was performed with regard to the spectrum of native chitosan (Figure 1D). The FTIR spectrum of chitosan (CH 85/500) showed the following characteristic bands corresponding to its functional groups: 3700–3000 cm^−1^—ν(O–H) overlapped with ν_s_(N–H); 2929 cm^−1^—ν_as_(C–H), 2881 cm^−1^—ν_s_(C–H); 1647 cm^−1^—ν(C=O) amide I; 1581 cm^−1^—δ_s_(NH_2_) amino group; 1410, 1321 cm^−1^—δ_s_(C–H) and δ(O–H); 1385 cm^−1^—δ_s_(C–H) amide group; 1264 cm^−1^—ν(C–O); 1198 cm^−1^—ν(N–H); 1150, 1059, 1026 cm^−1^—ν_as_(C–O–C) and ν_s_(C–O–C); 894 cm^−1^—ω(C–H); 660 cm^−1^—ω(N–H) [20,21,22]. The spectra of implants differing in concentration of chitosan displayed additional bands (Figure 1Da). The inner surfaces showed bands at 3640, 1424, and 871 cm^−1^. Moreover, a disappearance of bands at 1385 and 1321 cm^−1^ could be noted in relation to the spectrum of native chitosan. The outer surfaces showed broad bands at around 1398 (with the minimum moving toward lower wavenumbers for higher chitosan concentration) and 859 cm^−1^. Moreover, bands at 1385 and 1321 cm^−1^ could be noted at the right shoulder of band with the maximum at 1398 cm^−1^.

In addition to FTIR, ToF-SIMS analysis was used to identify the elemental and molecular composition of the inner and outer surfaces of deposits. It is worth noting that some parts of the signals coming from the surface of the studied materials could not be unequivocally assigned due to peak overlapping resulting from the irregular shape of the analyzed samples, which were additionally nonconductive. Therefore, the main focus was on the light fragmentation ions ascribed to main components of the investigated deposits, i.e., chitosan and hydroxyapatite. Such an approach allowed comparing the contribution of the studied native components in inner and outer surfaces of the obtained tubular-shaped structures. Table 3 lists the selected positive and negative ions observed on the secondary ion mass spectra of chitosan, hydroxyapatite, and the deposits. ${\mathrm{NH}}_{4}^{+}$ (m/z = 14) and ${\mathrm{CH}}_{4}N^{+}$ (m/z = 30) ions were selected as the representative ions of chitosan, which is composed of randomly distributed d-glucosamine (molecular mass 179) and N-acetyl-d-glucosamine (molecular mass 221) units [23,24]. The intensity for these ions was high for CH and low for HAp. Analyzing deposit samples obtained from chitosan–hydroxyapatite solutions differing in concentration of CH, a higher intensity of ${\mathrm{NH}}_{4}^{+}$ and ${\mathrm{CH}}_{4}N^{+}$ was observed with increasing concentration of chitosan. The intensity of ${\mathrm{NH}}_{4}^{+}$ and CH_4_N^+^ for inner surfaces was equal to 3.7 × 10^−3^ and 1.6 × 10^−2^ for 0.2 wt./vol.% CH and 6.4 × 10^−3^ and 2.7 × 10^−2^ for 0.8 wt./vol.% CH, respectively. The positive and negative ions characteristic for the HAp reference material identified in the presented work were ${\mathrm{Ca}}^{+}$ (m/z = 40), ${\mathrm{CaOH}}^{+}$ (m/z = 57), ${\mathrm{PO}}_{2}^{-}$ (m/z = 63), and ${\mathrm{PO}}_{3}^{-}$ (m/z = 79) [25,26]. The intensity of these ions was high for HAp and low for CH. The samples obtained from chitosan–hydroxyapatite solutions with a lower concentration of CH were characterized by lower intensities of ${\mathrm{Ca}}^{+}$ (m/z = 40), ${\mathrm{CaOH}}^{+}$ (m/z = 57), ${\mathrm{PO}}_{2}^{-}$ (m/z = 63), and ${\mathrm{PO}}_{3}^{-}$ (m/z = 79) compared to those obtained from chitosan–hydroxyapatite solutions with a higher concentration of CH.

In order to determine the types of interactions created in the process of electrodeposition among chitosan, lactic acid, and hydroxyapatite, the solutions differing in concentration of lactic acid and hydroxyapatite were examined.

The images of implants prepared from chitosan–hydroxyapatite solutions differing in concentration of lactic acid (i.e., maintaining a constant concentration of chitosan and hydroxyapatite) are shown in Figure 1Ab. A higher lactic acid concentration led to a lower mass of deposit (Figure 1Bb) and a lower water content (Figure 1Cb). The FTIR spectrum of lactic acid showed characteristic absorption bands at 1733 (CO double bound stretch characteristic for carboxylic acid group) and 1415 cm^−1^ (C–H bend) (Figure 1Da). The bands at 1226, 1126, and 1040 cm^−1^ were assigned to C–C and C–O stretching [27]. None of the studied inner surfaces showed bands around 1733 cm^−1^ (Figure 1Db). Analyzing the spectra of outer surfaces, only the surface with the higher lactic acid concentration used demonstrated a weak band with a maximum at 1744 cm^−1^. ToF-SIMS analysis showed that electrodeposition from the solution with a higher concentration of LA resulted in a higher intensity of ion identities characteristic for CH (${\mathrm{NH}}_{4}^{+}$ and ${\mathrm{CH}}_{4}N^{+}$) and a lower intensity of ion identities characteristic for HAp (${\mathrm{Ca}}^{+}$, ${\mathrm{CaOH}}^{+}$, ${\mathrm{PO}}_{2}^{-}$, and ${\mathrm{PO}}_{3}^{-}$). In addition, a difference in the intensity of these ions could be noted between the inner surfaces and outer surfaces of the analyzed structures.

Comparing the images of implants prepared from chitosan–hydroxyapatite solutions differing in concentration of hydroxyapatite (i.e., maintaining a constant concentration of chitosan and lactic acid, Figure 1Ac), it can be indicated that a higher concentration of hydroxyapatite led to a higher mass of deposit (Figure 1Bc) and a higher water content (Figure 1Cc). This observation could be attributed to the increasing content of hydroxyapatite-derived species in the deposited structures. Deposit porosity was lower for lower hydroxyapatite content (Figure 2B,C), which is in agreement with the water content studies. Native hydroxyapatite showed characteristic absorption bands at 1022, 962, 562, and 472 cm^−1^ which could be attributed to $\nu_{3}\left({\mathrm{PO}}_{4}^{3-}\right)$, $\nu_{1}\left({\mathrm{PO}}_{4}^{3-}\right)$, $\nu_{4}\left({\mathrm{PO}}_{4}^{3-}\right)$, and $\nu_{2}\left({\mathrm{PO}}_{4}^{3-}\right)$, respectively (Figure 1Da) [28]. The bands at 1414 and 889 cm^−1^ could be assigned to ${\mathrm{CO}}_{3}^{2-}$, which is often present in hydroxyapatite as a production residue [29]. Bands at 3633 cm^−1^ were characteristic for the stretching mode of the OH bond. The FTIR spectrum of the inner surface of implant prepared from the solution with a concentration of HAp equal to 0.025 wt./vol.% showed bands characteristic for chitosan (Figure 1Dc). The absorption bands at 1581 and 1377 cm^−1^ were more pronounced. Moreover, bands at 3643 and 874 cm^−1^ were weak. By contrast, the corresponding spectrum of sample prepared from the solution with a higher concentration of HAp (0.1 wt./vol.%) showed a wide absorption band with a maximum at 1406 cm^−1^. Moreover, it indicated well-pronounced narrow bands at 3639 and 873 cm^−1^. The outer surface of structures prepared from the solution with a concentration of HAp equal to 0.025 wt./vol.% showed characteristics similar to the spectrum of chitosan. Only the band at 1581 cm^−1^ was more pronounced. The corresponding spectrum of the sample prepared from the solution containing 0.1 wt./vol.% of HAp showed a wide absorption band with a maximum at 1377 cm^−1^ and a narrow one at 869 cm^−1^. Analyzing the results obtained by ToF-SIMS for samples prepared from solutions with different concentrations of HAp, it could be noted that there was no difference in intensity of ${\mathrm{Ca}}^{+}$ and ${\mathrm{CaOH}}^{+}$. Only the outer surface of the sample prepared from the solution with a lower concentration of HAp showed a slightly lower intensity of ${\mathrm{CaOH}}^{+}$ secondary ions. That for 0.025 HAp was equal to 1.8 × 10^−2^ and that for 0.1 HAp was equal to 2.5 × 10^−2^. Analyzing the signals obtained for ${\mathrm{PO}}_{2}^{-}$ and ${\mathrm{PO}}_{3}^{-}$, it could be noted that a higher concentration of HAp in solution led to higher intensities of the abovementioned ions. Moreover, there was a difference in the intensity of these secondary ions between the inner and outer surfaces of implants. The intensity of ${\mathrm{PO}}_{2}^{-}$ for the inner and outer surface was equal to 0.22 × 10^−3^ and 3.8 × 10^−3^ for 0.025 wt./vol.% HAp and 7.8 × 10^−3^ and 1.7 × 10^−2^ for 0.1 wt./vol.% HAp, respectively. That for ${\mathrm{PO}}_{3}^{-}$ for the inner and outer surface was equal to 1.6 × 10^−3^ and 1.9 × 10^−3^ for 0.025 wt./vol.% HAp and 5.5 × 10^−3^ and 9.3 × 10^−3^ for 0.1 wt./vol.% HAp, respectively.

The images of implants prepared from chitosan–hydroxyapatite solutions with the application of different voltages are shown in Figure 1Ad. A higher voltage led to a higher mass of deposit (Figure 1Bd) and a lower water content (Figure 1Cd). It could be noted that there was a minimum value of voltage allowing the course of electrophoretic deposition from chitosan–hydroxyapatite solution. No deposit was obtained at 3 V. The FTIR spectrum of the inner surface of the implant prepared by applying voltage equal to 6 V showed bands characteristic for chitosan (Figure 1Dd). The absorption bands at 1581 and 1377 cm^−1^ were more pronounced. By contrast, the spectrum of the sample prepared at 18 V showed a wide absorption band with a maximum at 1418 cm^−1^ and well-pronounced bands at 3638 and 872 cm^−1^. The spectrum of the outer surface of the sample obtained at 6 V resembled that of chitosan. The absorption bands at 1579 and 1375 cm^−1^ were more pronounced. The sample obtained at 18 V showed a wide absorption band with a maximum at 1388 cm^−1^ and well-pronounced bands at 3638 and 871 cm^−1^.

The images of implants prepared from chitosan–hydroxyapatite solutions with the application of different electrophoretic deposition times are shown in Figure 1Ae. The structures obtained after 10 and 30 min are shown in Figure 2A,E. A longer time of deposition led to a higher mass of deposit (Figure 1Be) and a lower water content (Figure 1Ce). The inner surface of implant prepared after 5 min showed bands characteristic for chitosan (Figure 1De). The absorption bands at 1578 and 1415 cm^−1^ were more pronounced. The spectrum of the outer surface of the implant showed similar characteristics with absorption bands at 1573 and 1379 cm^−1^. The spectra of the inner and outer surfaces of samples prepared after 20 min showed wide absorption bands with maxima at 1418 and 1400 cm^−1^, respectively. They also showed well-pronounced bands at 3638 and 872 cm^−1^.

## 4. Discussion

Chitosan can be dissolved in some diluted acids by protonation of its amino groups. This results in positively charged polymer chains.

(2) $$\mathrm{Chit}-{\mathrm{NH}}_{2}\;+{\;H}_{3}O^{+}\to \mathrm{Chit}-{\mathrm{NH}}_{3}^{+}\;+{\;H}_{2}O.$$

However, it should be taken into account that the solubility of chitosan depends on the value of pK_a_ of acid and its concentration [13]. It is soluble in dilute acidic solutions below pH = 6.0–6.5 [14,30]. When pH exceeds 6.0, the protonated amino groups release protons, consequently increasing the hydrophobicity along polymer chains.

In the process of electrophoretic deposition, the applied electric current causes motion of the charged chitosan and results in an accumulation of chitosan macromolecules at the cathode. As a consequence, an insoluble chitosan film is deposited on the cathode surface [16]. The most common interactions between biopolymer macromolecules created during deposition are hydrogen bonding and electrostatic interactions. Hydrogen bonds have a predominant impact on the structure and properties of biopolymers. Moreover, in solution, the charge of the chitosan macromolecule depends on its deacetylation degree (DA) and negatively charged counterions [31].

At the cathode, the following reaction takes place [16]:

(3) $$2H_{2}O\;+\;2e^{-}\to H_{2}\;+\;2{\mathrm{OH}}^{-}.$$

It results in a neutralization of the positively charged chitosan and the formation of an insoluble chitosan deposit on the cathode surface.

(4) $$\mathrm{Chit}-{\mathrm{NH}}_{3}^{+}\;+{\;\mathrm{OH}}^{-}\to \mathrm{Chit}-{\mathrm{NH}}_{2}\;+{\;H}_{2}O.$$

In this work, the higher chitosan concentration applied resulted in a higher mass of deposit (Figure 1Ba). Analyzing the structural characteristics of chitosan, a higher molecular weight of chitosan chains led to a higher mass of deposit and a higher porosity. Electrodeposition of chitosan macromolecule results from its neutralization upon creation of$\;{\mathrm{OH}}^{-}$ ions at the vicinity of the cathode (Equations (3) and (4)). Neutralization of polymer with a higher molecular weight might result in a higher mass of the deposit. Moreover, chitosan with a higher molecular weight might attract more hydroxyapatite-derived species; therefore, a higher mass of deposit is gained. The chitosan deacetylation degree had an insignificant influence on the resulting deposit mass (Figure 1Ba); however, the water content decreased with increasing deacetylation degree. The opposite phenomenon was observed for lactic acid. A lower pH led to a lower mass of deposit (Figure 1Bb).

The relationship among the linear charge density parameter (ξ), the dissociation degree (α), and the equivalent conductance (ε) can be expressed as

(5) $$\xi =1.38\mathrm{DA}\alpha ≅0.27\frac{\varepsilon }{\varepsilon_{0}},$$

where $\varepsilon_{0}$ is the limit equivalent conductance, which can be obtained from the variation of conductivity versus concentration $\left(C^{0.5}\right)$ [31]. The conductivity of the chitosan solution decreases with increasing pH. In solutions below pH 5, the high charge density of the linear chitosan chain causes development of a semirigid rod conformation. A higher conductivity is observed due to the higher concentration of charged particles. Upon raising the pH, amino groups undergo deprotonation and become accessible for hydrogen bonding. In dilute solutions, chitosan forms a small number of large sized aggregates. In these conditions, chitosan chains form the bulky helical structure, which is responsible for the reduction in their mobility by enhancing the drag force and by trapping acid anions inside the helix. It reduces the net charge (lower conductivity). The less protonated chitosan-based aggregates at a higher pH are more susceptible to neutralization by pH variation at the cathode surface, resulting in the relatively fast deposition of the insoluble chitosan film.

It is known that, in the range of pH values between the pKa of lactic acid (3.8) and that of chitosan (~6), an ionic pair between RCOO^−^ and protonated chitosan ($-{\mathrm{NH}}_{3}^{+}$) is formed [32]. It was shown that the lactic acid counter-anion interacts strongly with the chitosan chains [33]. Moreover, Cho and colleagues performed detailed studies on the influence of ionic strength on chitosan-based solutions [34]. They showed that the ionic strengths can be calculated from the following equation:

(6) $$I=\frac{1}{2}\sum \left(c_{i}Z_{i}^{2}\right),$$

where c_i_ and Z_i_ are the concentration and the charge number of ion i, respectively. With increasing lactic acid concentration, increasing ionic strengths will be observed. The ionic strength can be used to calculate the Debye screening length (k^−1^).

(7) $$k^{-1}={\left(\frac{1000{\varepsilon k}_{B}T}{8{\pi e}^{2}N_{\mathrm{Av}}I}\right)}^{0.5}=\frac{0.3043}{I^{0.5}},$$

where ε is the permittivity of water, k_B_ is the Boltzmann constant, T is the absolute temperature (K), e is the Coulomb electronic charge, and N_Av_ is the Avogadro number. The Debye screening length is a measure of the distance over which an individual charged particle exerts an electrostatic effect. From the above equation, the electrostatic double-layer thickness decreases inversely as the square root of the ionic strength. This decrease can be related to the screening of the positively charged amino groups of chitosan by anions (e.g., ${\mathrm{CH}}_{3}\mathrm{CH}\left(\mathrm{OH}\right){\mathrm{CO}}_{2}^{-}$ in the case of lactic acid). In consequence, a strong diminution of the repulsive potential can be observed. At the same time, the repulsion reduction can result in an increased risk of flocculation or precipitation. Such a phenomenon was observed earlier for chitosan solutions at I > 0.46 M (k^−1^ < 0.45 nm) [34]. A higher concentration of lactic acid leads to a lower concentration of the ${\mathrm{NH}}_{3}^{+}$ groups of chitosan chains in solution neutralized near the cathode. Therefore, a reduction in deposit mass with increasing lactic acid concentration was observed. This conclusion was also in agreement with the results of FTIR analysis. None of inner surfaces showed bands around 1733 cm^−1^. Analyzing the spectra of outer surfaces, only the surface with the highest lactic acid concentration applied demonstrated a weak band with a maximum at 1744 cm^−1^. Results obtained using ToF-SIMS for samples prepared from solutions with different concentrations of LA indicated that a higher pH of solution led to a higher intensity of ion identities characteristic for chitosan (Table 3). Oppositely, the signals indicating the presence of ions characteristic for hydroxyapatite (${\mathrm{Ca}}^{+}$, ${\mathrm{CaOH}}^{+}$, ${\mathrm{PO}}_{2}^{-}$, and ${\mathrm{PO}}_{3}^{-}$) were weaker.

Comparing images of implants prepared from chitosan–hydroxyapatite solutions differing in concentration of hydroxyapatite (i.e., maintaining a constant concentration of chitosan and lactic acid), it can be indicated that more hydroxyapatite led to a higher mass of deposit (Figure 1Bc). Neither the FTIR spectra of inner surfaces nor those of outer surfaces showed clear absorption bands characteristics for native hydroxyapatite [28]. In order to identify groups characteristic for hydroxyapatite-derived species, ToF-SIMS was applied (Table 3). Intensities of ions originating from HAp for samples prepared from solutions containing a higher concentration of hydroxyapatite were more pronounced than those for samples obtained from solutions with a lower concertation of hydroxyapatite. FTIR spectra showed bands at 1414 and 889 cm^−1^. These absorption bands could be assigned to ${\mathrm{CO}}_{3}^{2-}$, which is often present in hydroxyapatite as a production residue [29]. In the literature, it was reported that chitosan could adsorb metal ions well through chelation by the amide groups on glucosamine [35]. Furthermore, it has been assumed that ${\mathrm{Ca}}^{2+}$ would be preferably attached to the surface of the deposited chitosan backbone, forming nucleation sites for the growth of crystalline ${\mathrm{CaCO}}_{3}$ [36]. Upon a continuous local electrochemically induced pH increase around the cathode, deposition of chitosan takes place. Chitosan precipitation is accompanied by the production of dissociative anions of ${\mathrm{CO}}_{3}^{2-}$ in the vicinity of the cathode (from ${\mathrm{HCO}}^{3-}$ to ${\mathrm{CO}}_{3}^{2-}$, pK_a2_ ~ 10.3). When the supersaturation exceeds the critical level required for ${\mathrm{CaCO}}_{3}$ nucleation, it would precipitate onto the cathode. Thus, the cathodic deposition of chitosan (Equation (4)) is accompanied by the precipitation of ${\mathrm{CaCO}}_{3}$.

(8) $${\mathrm{Ca}}^{2+}+{\mathrm{HCO}}_{3}{}^{-}\;+{\mathrm{OH}}^{-}\to {\mathrm{CaCO}}_{3}\;+{\;H}_{2}O.$$

In addition, it is assumed that two kinds of sites exist during the electrodeposition at the cathode surface. One of them is for the precipitation of chitosan and ${\mathrm{CaCO}}_{3}$, and the other is for the generation of hydrogen gas bubbles. The sites where H_2_ bubbles are generated will not undergo deposition during the entire process. As a result, a porous structure is obtained.

The bands at 3633 cm^−1^ observed in FTIR spectra were characteristic for the stretching mode of the OH bond. They can result from the presence of $\mathrm{Ca}{\left(\mathrm{OH}\right)}_{2}$. Moseke and colleagues [37] showed that $\mathrm{Ca}{\left(\mathrm{OH}\right)}_{2}$ appears as a precursor for calcium phosphate formation during the initial phase of the precipitation process at lower pH values, but is replaced by brushite or hydroxyapatite with the current-induced increase in pH value due to the formation of hydrogen gas at the cathode and the remaining ${\mathrm{OH}}^{-}$ ions.

In order to explain the influence of time and voltage, Faraday’s law should be considered. It states that the deposition rate (w) is proportional to the quantity of electric charge (Q) passed through an electrochemical cell [38].

(9) w=ZQ,

where Z is the electrochemical equivalent, the constant of proportionality. Q is the product of the current (I) and elapsed time (t),

(10) Q=It;

thus,

(11) w=ZIt.

In this work, a longer time or higher voltage applied led to a higher deposition rate. These results are in agreement with Faraday’s law. Moreover, during the process of deposition, cathodic reduction of water and production of hydrogen gas at the cathode surface are observed. Therefore, when the growth rate was relatively low, a dense film was formed. However, at high growth rates, the gas entrapment in the growing film caused substantial pore formation.

On the basis of the obtained results, it can be concluded that the chemical and physical properties of the deposit fabricated in the process of electrodeposition from chitosan and hydroxyapatite solution can be controlled by both the initial concentration of solution components (i.e., chitosan, hydroxyapatite, and lactic acid) and the process parameters (i.e., time and voltage). The existing solutions for fabrication of implants for regeneration or replacement of tubular-shaped tissues and organs (e.g., three-dimensional (3D) printing, electrospinning, salt leaching) are usually performed at high temperatures and use substances that are inappropriate for biologically active substances [39,40,41,42]. The fact that electrodeposition can be conducted at room temperature and over a short time makes this process competitive for these solutions. In addition, the electrodeposition solution can be easily enriched in biologically active substances, e.g., in the form of polymer microsphere carriers. Then, these carriers can be deposited simultaneously with chitosan and hydroxyapatite-derived species.

## 5. Conclusions

The application of an electric current to a chitosan hydroxyapatite solution leads to the co-deposition of chitosan chains and hydroxyapatite-derived species (e.g., ${\mathrm{CaCO}}_{3}\;$and $\mathrm{Ca}{\left(\mathrm{OH}\right)}_{2}$). The deposition process is accompanied by cathodic reduction of water and production of hydrogen gas at the cathode surface. All studied parameters, i.e., initial concentration of solution components (i.e., chitosan, lactic acid, and hydroxyapatite) and process parameters (i.e., time and voltage), had an influence on the resulting mass and structural properties of deposit. Therefore, by manipulating them, it is possible to adjust the deposit properties to the needs of the field of application. It is anticipated that the studied tubular-shaped hydrogel structures might be used in the regeneration or replacement of cylindrical tissues and organs.

## Figures and Tables

**Table 1:** Degree of deacetylation (DD), viscosity (µ), and viscosity average molecular weight (Mv) of polymers.

Polymer	DD (%)	µ (mPas)	Mv (kDa)
CH 85/100	82.6–87.5	71–150	309
CH 85/500	82.6–87.5	351–750	472
CH 85/1000	82.6–87.5	751–1250	738
CH 75/500	72.6–77.5	351–750	689
CH 95/500	≥92.6	351–750	237

**Table 2:** The studied parameters influencing deposition from chitosan (CH)–hydroxyapatite (HAp) solution. LA, lactic acid.

Parameter	C_CH_ (wt./vol.%)	C_LA_ (wt./vol.%)	C_HAp_ (wt./vol.%)	*U* (V)	*t* (min)
CH concentration	0.2, 0.4, 0.6, 0.8	3	0.1	12	10
LA concentration	0.4	2, 3, 4	0.1	12	10
HAp concentration	0.8	3	0.025, 0.05, 0.075, 0.1	12	10
voltage	0.8	3	0.1	3, 6, 12, 18	10
time	0.8	3	0.1	12	5, 10, 15, 20

**Table 3:** Intensity of selected positive and negative secondary ions collected from chitosan (CH 85/500), hydroxyapatite, and surfaces of tubular-shaped chitosan–hydroxyapatite deposits. The solution concentrations are listed in Table 2.

Material/Deposit	$NH_{4}^{+}$	$CH_{4}N^{+}$	$Ca^{+}$	$CaOH^{+}$	$PO_{2}^{-}$	$PO_{3}^{-}$
CH	1.2 × 10^−2^	4.7 × 10^−2^	1.8 × 10^−3^	6.8 × 10^−3^	0.8 × 10^−3^	1.6 × 10^−3^
HAp	0.6 × 10^−3^	3.0 × 10^−3^	2.1 × 10^−1^	1.4 × 10^−1^	3.2 × 10^−1^	2.8 × 10^−1^
0.2 CH inner/outer	3.7 × 10^−3^	1.6 × 10^−2^	9.3 × 10^−3^	2.4 × 10^−2^	2.9 × 10^−3^	2.1 × 10^−3^
0.8 CH inner/0.1 HAp inner	6.4 × 10^−3^	2.7 × 10^−2^	1.2 × 10^−2^	2.7 × 10^−2^	7.8 × 10^−3^	5.5 × 10^−3^
0.8 CH outer/0.1 HAp outer	5.8 × 10^−3^	2.5 × 10^−2^	1.2 × 10^−2^	2.5 × 10^−2^	1.7 × 10^−2^	9.3 × 10^−3^
0.025 HAp inner	5.4 × 10^−3^	2.7 × 10^−2^	1.2 × 10^−2^	2.6 × 10^−2^	2.2 × 10^−3^	1.6 × 10^−3^
0.025 HAp outer	5.5 × 10^−3^	2.7 × 10^−2^	1.1 × 10^−2^	1.8 × 10^−2^	3.8 × 10^−3^	1.9 × 10^−3^
2 LA inner	4.0 × 10^−3^	1.8 × 10^−2^	5.6 × 10^−2^	5.5 × 10^−2^	1.4 × 10^−2^	1.0 × 10^−2^
2 LA outer	4.2 × 10^−3^	1.8 × 10^−2^	3.7 × 10^−2^	5.3 × 10^−2^	1.6 × 10^−2^	1.1 × 10^−2^
4 LA inner	7.2 × 10^−3^	3.1 × 10^−2^	2.5 × 10^−2^	4.7 × 10^−2^	1.0 × 10^−3^	1.1 × 10^−3^
4 LA outer	6.8 × 10^−3^	2.9 × 10^−2^	1.7 × 10^−2^	4.1 × 10^−2^	9.5 × 10^−3^	7.2 × 10^−3^

## Data Availability

The data presented in this study are available on request from the corresponding author.
